# The Cytotoxic Effects of Geranylgeranylacetone Are Attenuated in the High-Glucose Condition

**DOI:** 10.1089/biores.2018.0041

**Published:** 2019-10-25

**Authors:** Yuko Nakano, Daisuke Kobayashi, Masao Miyake, Ryoko Kanno, Masahiro Murakawa, Akihiro Hazama

**Affiliations:** ^1^ Department of Anesthesiology, School of Medicine, Fukushima Medical University, Fukushima, Japan.; ^2^ Department of Cellular and Integrative Physiology, School of Medicine, Fukushima Medical University, Fukushima, Japan.

**Keywords:** cytotoxicity, geranylgeranylacetone, HeLa, Caco-2, HEK293

## Abstract

Geranylgeranylacetone (GGA) has been used as an antiulcer drug and also is known as inducer of heat shock protein 70 that has cytoprotective effects especially in hyperglycemic condition. In contrast, cytotoxicity of GGA has also been reported. Some studies have reported that GGA suppresses cell growth and induces apoptosis in cell models of human leukemia, ovarian carcinoma, and colon cancer *in vitro*. Therefore, the aim of this study was to determine whether GGA can have a cytotoxic effect on a human cervical cancer cell line (HeLa), human colorectal adenocarcinoma cells (Caco-2), and human embryonic kidney cells 293 (HEK) in normal-glucose and high-glucose environments (NG and HG, respectively). The results showed that 100 μM GGA inhibited proliferation of HeLa cells only in NG environment despite inhibiting proliferation of Caco-2 and HEK cells regardless of glucose concentration. Cell viability assay revealed that GGA decreased viability of HeLa, Caco-2, and HEK cells only in NG environment. Flow cytometric analyses revealed that the type of cell death was a combination of necrosis and apoptosis. Our study revealed that difference in cytotoxicity of GGA is influenced by glucose condition. The cytotoxic effects of GGA are attenuated in the HG condition. Since both cytotoxic and cytoprotective effects are reported about GGA, further research is needed about the mechanism of the cytotoxic effects.

## Introduction

Oral administration of geranylgeranylacetone (GGA), acyclic isoprenoid compound, has been used in Asia, as an antiulcer drug for >20 years, and no major adverse effects have been reported. GGA is also known as the drug that activates heat shock protein 70 (HSP70) and exerts cytoprotective effects against various stressors in a variety of cells and tissues.^1–7^ The mechanism is thought that GGA preferentially binds to the C-terminal of HSP70 and the chaperone activity of HSP70 is suppressed, and subsequently HSP70 transcription activity is upregulated by an activation of transcriptional factor.^8^

Recently, it has also been reported that HSP70 is decreased in hyperglycemic humans.^9–11^ Notably, there is a report that administration of GGA induced HSP70 and improved glucose tolerance in diabetic monkeys.^12^ It is widely accepted that the endoplasmic reticulum (ER) stress that is closely related to the production of molecular chaperones including HSP70 in diabetes causes insulin resistance,^13–16^ and cytoprotective effects of GGA in hyperglycemic condition have been focused in these years.

In contrast, several studies reported that isoprenoid compound and its derivative compounds, such as docetaxel and pacritaxel, had an anticancer effect and were currently available for clinical use.^17–20^ Moreover, Iwao and Shidoji^21^ focused on geranylgeranoic acid, the isoprenoid compound produced *in vivo*, and showed that it has antitumor effects in human hepatoma cells. They revealed that cell death caused by geranylgeranoic acid is due to induction of nuclear translocation of mutant cytoplasmic p53, which is not a typical apoptotic process, but nonnecrotic cell death.^22–24^ GGA has been shown to induce an apoptosis, and cause of proliferation and viability reduction in human leukemia cells (ML1, U937, HL60, and K562),^25,26^ ovarian carcinoma cells (Caov-3 and SKOV-3),^27,28^ and melanoma cells (G361, SK-MEL-2, and SK-MEL-5).^29^ Furthermore, a recent study reported that GGA inhibits proliferation of human colon cancer cells (DLD-1 and HT29) by inducing apoptosis and cell cycle arrest.^30^

Although GGA has several reports about cytotoxic effects, few reports have evaluated cytotoxicity at high-glucose (HG) condition. In this study, we aimed to investigate whether the effect of GGA differs depending on the glucose concentration on several cell lines. For this purpose, we evaluated the effect of GGA on human cervical cancer cells (HeLa), human colorectal adenocarcinoma cells (Caco-2), and human embryonic kidney (HEK) cells 293 proliferation in normal-glucose (NG) (5.6 mM, 100 mg/dL) and HG (25 mM, 450 mg/dL) conditions.

## Materials and Methods

### Cell culture and GGA treatment

HeLa, Caco-2, and HEK cells were cultured in Dulbecco's modified Eagle's medium (DMEM) supplemented with 10% fetal bovine serum, 200 μg/mL streptomycin, and 200 U/mL penicillin. Experiments were performed using overnight cell cultures in DMEM supplemented with either a HG (25 mM) or a NG (5.6 mM) concentration. The medium was changed with GGA treatment. GGA (Teprenone; Tokyo Chemical Industry Co., Ltd., Tokyo, Japan) was dissolved in dimethyl sulfoxide (DMSO) and <1% DMSO was added to each culture. Control cultures were treated with the vehicle (1% DMSO). Positive control cultures of apoptosis were treated with staurosporine (4 μM) and were then incubated for 8 h.

### Lactate dehydrogenase and cell viability assay

HeLa, Caco-2, and HEK cells cultured in 96-well culture plates were assessed. Lactate dehydrogenase (LDH) activity was measured using a Cytotoxicity LDH Assay Kit-WST (Dojindo Laboratories, Kumamoto, Japan), according to the manufacturers' instructions. Cell viability was also evaluated using the trypan blue dye exclusion assay. In total, 1.0 × 10^5^ HeLa cells were seeded in dishes (35 mm in diameter) and treated with 100 μM GGA concentrations. As a control, the vehicle alone was assessed. After addition of 100 μM GGA to culture medium, cells were incubated for 1–3 days, then the cells were detached and mixed with an equal volume of a 0.4% trypan blue solution. The number of total and live cells was counted by TC20™ Automated Cell Counter (Bio-Rad Laboratories, Inc., Hercules, CA).

### Detection of apoptosis by flow cytometry

HeLa cells were treated with 100 μM GGA for 24 h and then analyzed by flow cytometry. The cells were detached by trypsin/ethylenediaminetetraacetic acid (EDTA) solution and suspended in each experimental solution before flow cytometric measurements. During early apoptosis, membrane integrity was assessed by staining with annexin V, which binds to exposed membrane phosphatidylserine, an acidic (anionic) phospholipid and one of the earliest features of apoptosis, using the PE Annexin V Apoptosis Detection Kit (BD Biosciences, Franklin Lakes, NJ). Necrotic changes were concurrently assessed using 7-amino-actinomycin D (7-AAD) staining solution to exclude viable cells with intact membranes.

### Statistical analysis

Statistical analysis was performed using SPSS statistical software (ver. 17; SPSS, Inc., Chicago, IL). The unpaired Student's *t*-test was used to determine statistical significance. Differences at a *p* value <0.05 were considered statistically significant.

## Results

### GGA cytotoxicity on cell culture

**FIG. 1.**
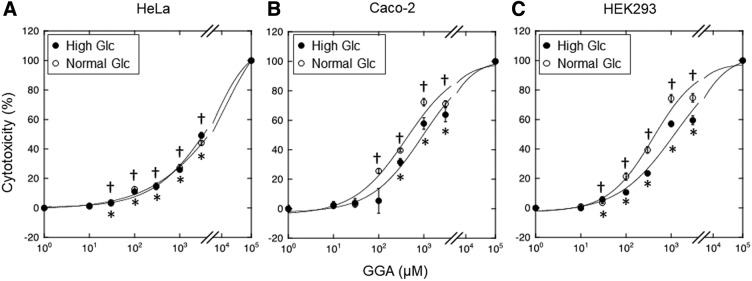
GGA cytotoxicity on HeLa **(A)**, Caco-2 **(B)**, and HEK293 **(C)** cells. The cytotoxicity under the HG and NG conditions assessed using LDH assay is shown. All data are presented as means ± SDM (vertical bar) and an asterisk indicates statistical significance in HG, and a dagger indicates statistical significance in NG compared with control (*p* < 0.05 using Dunnet's test). GGA, geranylgeranylacetone; HG, high glucose; LDH, lactate dehydrogenase; NG, normal glucose; SDM, standard difference in means.

The cytotoxicity of GGA on HeLa, Caco-2, and HEK cells was assessed using LDH assay (Fig. 1). Inhibitory concentration 50 of GGA on HeLa, Caco-2, and HEK cells was 3.6 ± 0.86 mM, 1.3 ± 0.47 mM, and 1.0 ± 0.32 mM in HG condition, and 4.4 ± 1.5 mM, 0.43 ± 0.11 mM, and 0.44 ± 0.15 mM in NG condition, respectively (Table 1). The cytotoxicity of HeLa and HEK cells was observed at >30 μM GGA, and that of Caco-2 cells was observed at >300 μM GGA in HG condition. In contrast, susceptibility of GGA to HeLa and Caco-2 cells increased in NG condition except for HeLa cells. There were no significant differences of cytotoxicity between HG and NG condition up to 1 mM GGA, and the cytotoxicity was higher at 3 mM GGA on HeLa cells. The cytotoxicity was higher at >100 μM GGA both in Caco-2 and HEK cell.

**Table 1.** Half Inhibitory Concentration of Geranylgeranylacetone on Several Cell Lines

	NG (mM)	HG (mM)
HeLa	4.4 ± 1.5	3.6 ± 0.86
Caco-2	0.44 ± 0.15	1.0 ± 0.32
HEK293	0.43 ± 0.11	1.3 ± 0.47

HG, high glucose; NG, normal glucose.

### GGA inhibited proliferation of HeLa, Caco-2, and HEK cells

**FIG. 2.**
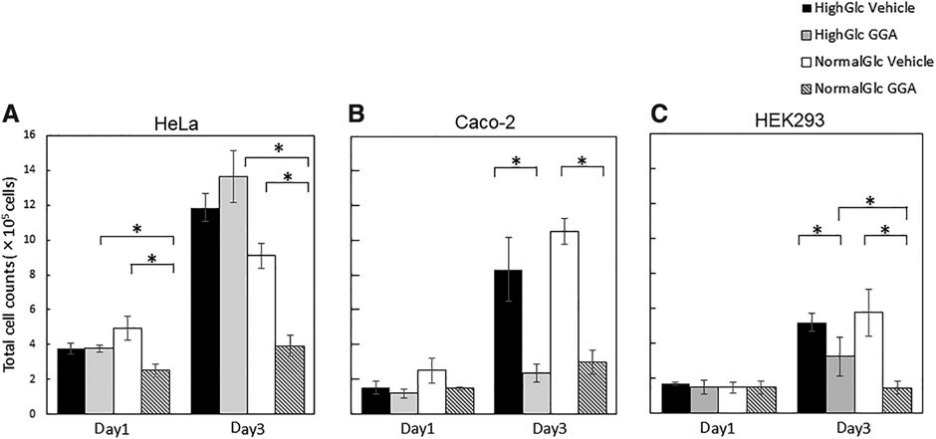
Effect of GGA on the cell proliferation of HeLa, Caco-2, and HEK293 cells. The total number of HeLa **(A)**, Caco-2 **(B)**, and HEK293 cells **(C)** is shown. The cells were cultured under HG (black and gray bar) and NG (white and hatched bar) in the presence (gray and hatched bar) and in the absence (black and white bar) of 100 μM GGA for 1 and 3 days. Data are presented as the means ± SDM (vertical bar). An asterisk indicates statistical significance (*p* < 0.05 using Student's *t*-test).

We counted total cells for 3 days after 0.1 mM GGA treatment in NG and HG conditions to evaluate the effect of GGA on the cell proliferation and viability of HeLa, Caco-2, and HEK cells. The data of days 1 and 3 are shown (Fig. 2). The total cells count decreased in GGA-treated cultures under NG conditions. The number of HeLa cells in the HG control and HG/GGA increased by ∼10-fold, whereas after 3 days of culture, the number of HeLa cells in the NG control and NG/GGA increased by ∼9-fold and 4-fold, respectively. The number of HeLa cells was 9.1 × 10^5^ cells in the NG control and 3.9 × 10^5^ cells in the NG/GGA after 3 days of culture. Total cell counts decreased by GGA treatment only in the NG condition.

In contrast, GGA inhibited the proliferation of HEK and Caco-2 after in both HG and NG conditions (Fig. 2). The number of HEK cells in the HG and NG control increased by ∼5-fold and 6-fold, respectively, whereas after 3 days of culture, the number of HEK cells in the HG/GGA and NG/GGA increased by ∼3-fold and 1.5-fold, respectively. The number of HEK cells was 5.8 × 10^5^ cells in the NG control and 1.5 × 10^5^ cells in the NG/GGA after 3 days of culture. The proliferation of Caco-2 cells was also suppressed in both HG/GGA and NG/GGA conditions. The number of Caco-2 cells in HG/GGA increased by approximately twofold, whereas that in the HG control increased by approximately eightfold. The number of Caco-2 cells in NG control and NG/GGA increased by ∼6-fold and 1.5-fold, respectively. The number of Caco-2 cells was 10.5 × 10^5^ cells in the NG control and 3.0 × 10^5^ cells in the NG/GGA after 3 days of culture.

### GGA decreased viability of HeLa, Caco-2, and HEK cells only in the NG environment

**FIG. 3.**
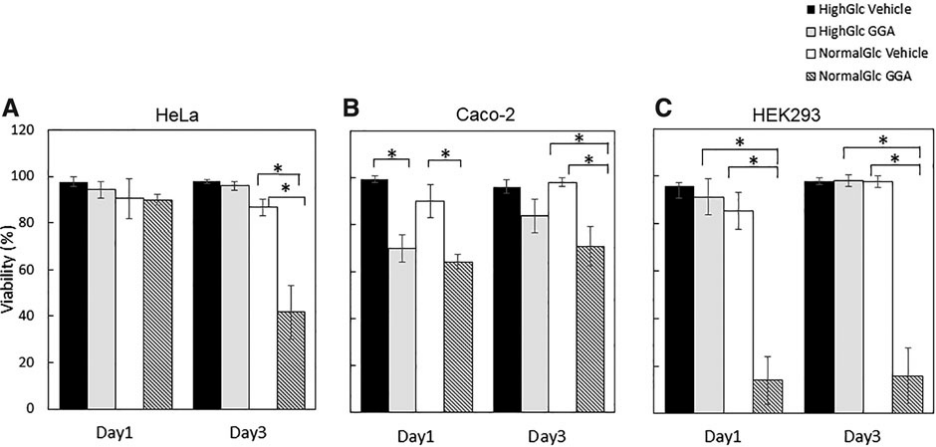
Effect of GGA on the cell viability of HeLa, Caco-2, and HEK293 cells. Cell viability of HeLa **(A)**, Caco-2 **(B)**, and HEK293 **(C)** is shown. The cells were cultured under HG (black and gray bar) and NG (white and hatched bar) in the presence (gray and hatched bar) and in the absence (black and white bar) of 100 μM GGA for 1 and 3 days. The data are presented as the means ± SDM (vertical bar). An asterisk indicates statistical significance (*p* < 0.05 using Student's *t*-test).

The percentage of viability decreased 3 days after GGA treatment only in the NG condition, whereas there was no remarkable change in the HG condition on HeLa, Caco-2, and HEK cells. The reduction was remarkable in the NG/GGA day 3 (Fig. 3). The percentage of viability in the NG/GGA reduced to 41.6% after 3 days treatment, whereas that in the HG/GGA was ∼90% in HeLa cells. The percentage of viability in the NG/GGA reduced to 15.1% after 3 days treatment, whereas that in the HG/GGA was 98.1% in HEK cells. The percentage of viability in the HG/GGA and NG/GGA was 83.6% and 70.7%, respectively, in Caco-2 cells.

### GGA induced apoptosis in NG environment on HeLa cells

**FIG. 4.**
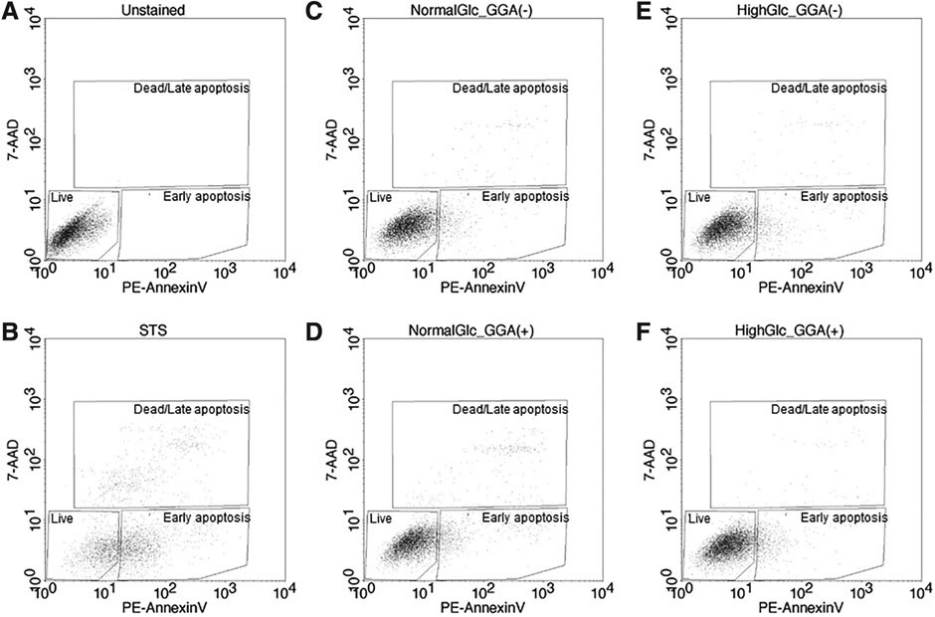
Apoptotic cell analysis by flow cytometry. Cell apoptosis was detected by both annexin V (*x*-axis) and 7-amino-actinomycin D (*y*-axis) staining. Unstained cells **(A)** were used as live cells gating. STS-treated cells **(B)** were used as early apoptosis and late apoptosis/dead cells gating. Cells were cultured under NG **(C, D)** and HG **(E, F)** in the absence **(C, E)** and in the presence **(D, F)** of 100 μM GGA. STS, staurosporine.

Evaluation of apoptotic cells was measured in HeLa cells by PE annexin V and 7-AAD staining in the NG and HG conditions with and without 100 μM GGA. As shown in Table 2 and Figure 4, the early apoptotic percentage of the NG/GGA (9.5 ± 0.4%) was higher than that of the HG/GGA (7.4 ± 0.1%). The late apoptotic and dead cell percentage of the NG/GGA (4.2 ± 0.7%) was also higher than that of the HG/GGA (0.8 ± 0.1%). The population of both the early apoptotic and late apoptotic/dead cells increased after GGA treatment in NG condition. In the case of HG condition, the population of the early apoptotic cells increased after GGA treatment, although there were no significant differences about late apoptotic/dead cells populations between the presence and the absence of GGA.

**Table 2.** Population of Apoptotic Cells in the Normal-Glucose and High-Glucose Conditions

	NG		HG		Staurosporine
	GGA (–)	GGA (+)	GGA (–)	GGA (+)	
	Mean ± SD		Mean ± SD		Mean ± SD
Live (%)	91.7 ± 0.9	84.8 ± 0.9^a,b^	92.9 ± 0.2	90.5 ± 0.4^a^	36.3 ± 1.3
Dead/late apoptosis (%)	1.0 ± 0.1	4.2 ± 0.7^a,b^	1.0 ± 0.2	0.8 ± 0.1	11.1 ± 0.3
Early apoptosis (%)	6.1 ± 0.7	9.5 ± 0.4^a,b^	5.1 ± 0.1	7.4 ± 0.1^a^	48.3 ± 1.0

^a^ Statistical significance between GGA (–) and GGA (+).

^b^ Statistical significance between NG and HG.

*p* < 0.05 using Student's *t*-test.

GGA, geranylgeranylacetone; SD, standard deviation.

## Discussion

This study demonstrates that GGA has cytotoxic effects, although the effects may be attenuated in the HG condition. We evaluated the effects of GGA in HeLa, Caco-2, and HEK cells in NG (5.6 mM, 100 mg/dL) and HG (25 mM, 450 mg/dL) conditions. Total cell counts revealed that GGA inhibited proliferation of HeLa cells only in NG environment despite inhibiting proliferation of Caco-2 and HEK cells regardless of glucose concentration. Cell viability assay revealed that GGA decreased viability of HeLa, Caco-2, and HEK cells only in NG environment.

The glucose environment is an important factor of homeostasis for maintaining the normal metabolism of cells, and abnormal glucose conditions induce cellular ER stress and unfolded protein response (UPR) that induce molecular chaperones or leading apoptosis.^31,32^ Healthy normal blood sugar ranges for adult humans are between 4.0 and 5.4 mM (72–99 mg/dL) when fasting.^33^ However, there are some situations in which NG environment is not maintained.

For example, an insufficient angiogenesis in solid tumors results in glucose starvation, chronic anoxia, and low pH conditions. The 78-kDa glucose-regulated protein (GRP78), one of the best-characterized ER chaperones, is induced depending on cellular microenvironment such as low glucose and hypoxia condition.^34^ The GRP78 is overexpressed in several kinds of human tumors such as breast cancer,^35^ prostate cancer,^36^ leukemia,^37^ hepatocarcinoma,^38^ and cervical cancer.^39^ It seems that the GRP78 is a positive inducer for chemoresistance acquisition in tumor tissues and cells.^39^ The UPR under low-glucose conditions is considered to make cancer cells survive in a stressful microenvironment and contribute to resistance to chemotherapies.^40^

In contrast, a typical pathological condition in which HG conditions persist is diabetes. It is known that persistence of high-fat diet or hyper glucose condition in diabetes causes ER stress that induces apoptosis of pancreatic beta cells and insulin resistance.^9,12,14,15^ Insulin-resistant and hyperglycemic people have reduced HSP70 protein and gene expression.^14–16^ A recent study reported that GGA administration has induced HSP70 and improved insulin sensitivity and glucose tolerance in monkeys.^10^

Although both cytoprotective and cytotoxic effects of GGA are reported, little is known about the mechanism of the cytotoxic effects of GGA. Yoshikawa et al. revealed in human colon cancer cells (DLD-1 and HT29) that GGA dose-dependently activated caspase-3, -8, and -9, which play central roles in the apoptotic cascade.^30^ They hypothesized that apoptosis was induced by two major caspase pathways. One was the death receptor pathway, which was involved in caspase-8, and the other was the mitochondrial pathway, in which various signals can trigger the release of harmful proteins by mitochondria into the cytoplasm, leading to the activation of caspase-9. Both pathways result in the downstream activation of caspase-3.^14^ In this study, both apoptotic and late apoptotic or necrotic cell death was induced by GGA. The results do not contradict to previous studies.^41,42^ In this study, less cytotoxicity was observed in HG condition. We hypothesized that the molecular chaperones such as HSP70 may be induced and cytoprotective effects of GGA work at the same time in HG condition. Verification of this hypothesis and the expression level in that case are for further study.

Although several studies reported about the antitumor effects of GGA, our study revealed that GGA was also cytotoxic to HEK as well as HeLa and Caco-2 cells under normal glucose condition. Since GGA is cytotoxic to both tumor cells and normal cells or its effect depends on tissue and cell level, it can be said that clinical application should be carefully considered.

In summary, we have shown the difference of effects of GGA in the HG and NG conditions. The cytotoxic effects of GGA are attenuated in the HG condition. Since both cytotoxic and cytoprotective effects are reported about GGA, further research is needed about the mechanism of the cytotoxic effects.

## Abbreviations Used

7-AAD: 7-amino-actinomycin D
DMEM: Dulbecco's modified Eagle's medium
DMSO: dimethyl sulfoxide
EDTA: ethylenediaminetetraacetic acid
ER: endoplasmic reticulum
GGA: geranylgeranylacetone
GRP78: 78-kDa glucose-regulated protein
HG: high glucose
HSP70: heat shock protein 70
LDH: lactate dehydrogenase
NG: normal glucose
SD: standard deviation
SDM: standard difference in means
STS: staurosporine
UPR: unfolded protein response
